# Sandwiched confinement of quantum dots in graphene matrix for efficient electron transfer and photocurrent production

**DOI:** 10.1038/srep09860

**Published:** 2015-05-21

**Authors:** Nan Zhu, Kaibo Zheng, Khadga J. Karki, Mohamed Abdellah, Qiushi Zhu, Stefan Carlson, Dörthe Haase, Karel Žídek, Jens Ulstrup, Sophie E. Canton, Tõnu Pullerits, Qijin Chi

**Affiliations:** 1 Department of Chemistry, Technical University of Denmark, Kemitorvet Building 207, DK-2800 Kongens Lyngby, Denmark; 2 Department of Chemical Physics, Lund University, Box 124, 22100, Lund, Sweden; 3 The MAX IV Laboratory, Lund University, Box 124, 22100, Lund, Sweden; 4 Department of Chemistry, Faculty of Science, South valley University, Qena 83523, Egypt

## Abstract

Quantum dots (QDs) and graphene are both promising materials for the development of new-generation optoelectronic devices. Towards this end, synergic assembly of these two building blocks is a key step but remains a challenge. Here, we show a one-step strategy for organizing QDs in a graphene matrix via interfacial self-assembly, leading to the formation of sandwiched hybrid QD-graphene nanofilms. We have explored structural features, electron transfer kinetics and photocurrent generation capacity of such hybrid nanofilms using a wide variety of advanced techniques. Graphene nanosheets interlink QDs and significantly improve electronic coupling, resulting in fast electron transfer from photoexcited QDs to graphene with a rate constant of 1.3 × 10^9^ s^−1^. Efficient electron transfer dramatically enhances photocurrent generation in a liquid-junction QD-sensitized solar cell where the hybrid nanofilm acts as a photoanode. We thereby demonstrate a cost-effective method to construct large-area QD-graphene hybrid nanofilms with straightforward scale-up potential for optoelectronic applications.

Colloidal nanocrystals are among the essential building blocks employed in design and architecture of advanced functional materials because of their ability to interface inorganic, organic and biological components^1^,^2^. Among many applications studied, the use of colloidal nanocrystals for the fabrication of electronic and optoelectronic devices is of particular interest^2^. A colloidal nanocrystal is typically composed of an inorganic particle core and a capping layer. When the inorganic core of the particle is a semiconductor, the nanocrystal is commonly termed quantum dot (QD)^2^. QDs exhibit strong quantum-size effects, leading to a sensitively size-tunable bandgap and high luminescence quantum yield^3^. These characteristics make them promising materials for a wide range of purposes^4^,^5^. For example, their impressive contributions to biomedical research in the past two decades is well documented^6^,^7^, and QDs have been crucial in the research and development of next-generation solar cells^8^,^9^,^10^,^11^,^12^,^13^,^14^,^15^. In the latter, QDs are recognized as a novel type of light harvesters due to their high extinction coefficient, versatile tunability, high stability, and capability of multiple exciton generation.

In a QD-sensitized solar power conversion system, the two key components are the QDs as sensitizer and the electrode as electron acceptor. Metal oxides (MO) such as TiO_2_ particles and ZnO nanowires have been the most commonly used electrode materials^10^,^11^. The overall efficiency of a solar power conversion system is controlled by a sequence of electronic events. Upon photoexcitation of the QDs, charge separation proceeds via interfacial electron transfer (ET) or hole transfer (HT) from QDs to the electrodes. The development of new electrode materials, synergic assembly of the two key components, and understanding of charge transport mechanisms are the central issues towards realizing optimal design and construction of high-performance QD based solar power conversion systems. Our recent efforts have been devoted to some of these fundamental aspects such as the ET mechanisms and key determinant factors in the systems consisting of CdSe QDs and ZnO nanowires^16^,^17^,^18^,^19^,^20^,^21^,^22^,^23^,^24^. In addition, we have offered a precise approach both theoretically and experimentally to directed energy transfer in thin films composed of QDs with different sizes^25^.

Graphene as a wonder material has emerged at an unprecedented pace over the past decade^26^,^27^,^28^. This material has continued to develop as an effective alternative to many conventional optoelectronic, photonic, plasmonic and energy materials. A recent report provides clear evidence for ultra-fast collinear scattering and carrier multiplication in graphene itself^29^. In contrast to its counterparts, the carbon nanotubes and fullerenes, the two-dimensional crystalline structure and high flexibility of graphene make it an ideal platform for the architecture of hetero-structured nanocomposites^30^ that can further improve light-matter interactions^31^. Graphene can promote the photocatalytic performance of semiconductors for solar energy conversion^32^, and combining chemically exfoliated graphene with QDs could produce a new kind of optoelectronic nanocomposite with high light-harvesting efficiency^33^,^34^,^35^,^36^,^37^. A state-of-the-art study recently by Konstantatos and co-workers has clearly demonstrated that hybrid graphene-QD films enable achieving an ultrahigh gain of electrons per photon^38^, which is expected to significantly promote the development of new-generation phototransistors based on hybrid QD-graphene materials. Furthermore, solution-processed routes offer a cost-effective and operation-flexible method for the preparation of thin-film based photovoltaic cells^39^. However, synergic assembly of QDs and graphene nanosheets remains a challenging issue. Our current efforts aim at optimizing assembly of QDs in a graphene matrix for highly-efficient electron transfer. In this work, we have demonstrated that two-phase solution processing enables simultaneous attachment and self-assembly of sandwiched QD-graphene hybrid nanofilms. The as-prepared hybrid films show fast photoinduced electron transfer and display significant enhancement of photocurrent generation compared to the films composed of pure QDs.

## Results

### Self-assembled construction of QD-graphene hybrid nanofilms

The building block materials, including graphene oxide (GO), reduced graphene oxide (RGO), and CdSe QDs were first synthesized and characterized. GO was prepared according to our previous procedure^40^,^41^ and characterized systematically. Single-layered GO suspensions were obtained and tested for two-phase reduction by hydrazine^40^ (Supplementary Figs. S1-S3). CdSe QDs (2.9 nm) were synthesized as reported^16^,^17^,^18^ and are featured by a strong absorption peak at 542 nm. The CdSe QD surface capped with oleic acid (OA) with a long alkyl chain is strongly hydrophobic (Supplementary Fig. S4) facilitating a uniform dispersion of such QDs in toluene.

**Figure 1:**
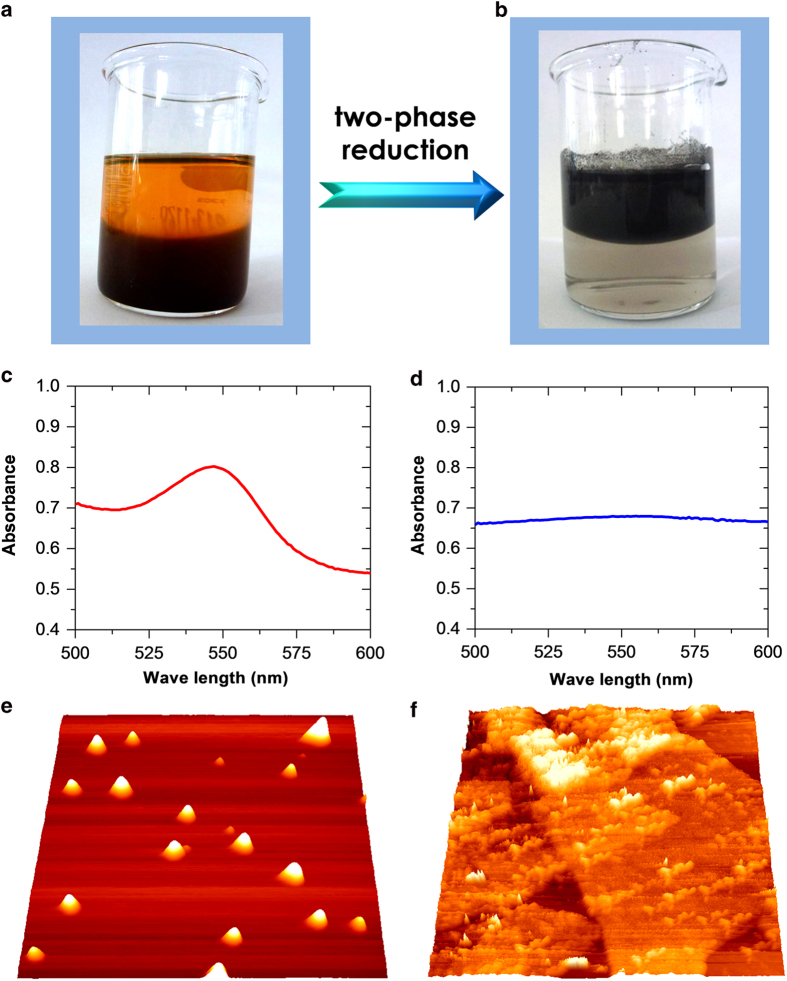
**Formation and microscopic characterization of QDs-RGO hybrid composite.** (**a**) and (**b**) Photographs of two-phase solutions before (**a**) and after (**b**) interfacial reduction of GO nanosheets. (**c**) and (**d**) UV-vis spectra of QDs in the organic phase before (**c**) and after (**d**) interfacial reduction. (**e**) and (**f**) 3D AFM images of QDs in isolated form (**e**) and adsorbed on RGO nanosheets (**f**). The scanned areas in the AFM images: (**e**) 1 μm x 1 μm; (**f**) 2.5 μm × 2.5 μm.

In a typical two-phase synthesis, we used toluene as the oil (O) phase and water (W) as the aqueous phase. Prior to heat-assisted reduction, CdSe QDs were dispersed solely in the toluene phase (the top layer in Fig. 1a), while GO nanosheets were located only in the aqueous solution (the bottom layer in Fig. 1a). For example, the initial absorbance of CdSe QDs at 542 nm is 0.25 (Fig. 1c). The water phase became almost colorless upon the reduction of GO to RGO by hydrazine, while the toluene phase color changed to dark brown. This is due to the attachment of CdSe QDs to RGO (Fig. 1b). The attachment of QDs to RGO nanosheets from the toluene phase is evidenced by a dramatic decrease of the 542 nm absorbance to 0.01 (Fig. 1d), i.e. only 4.6% CdSe QDs remained in the organic phase. The UV-vis spectra thus indicate that CdSe QDs from the toluene phase were strongly adsorbed onto the graphene nanosheet surface, driven by the hydrophobic interactions between the RGO planes and the capping ligand on the CdSe QDs. This is supported by AFM. High-resolution AFM images show that RGO nanosheets obtained by the O/W interface method have a lateral dimension of 1-2 μm with a height around 0.6-0.8 nm (Supplementary Fig. S2). AFM images of CdSe QDs (e.g., Fig. 1e) show an average height of ca. 3 nm, consistent with the TEM results (on average 2.9 nm). Once CdSe QDs met graphene nanosheets at the O/W interfaces, they were adsorbed on the graphene nanosheet surface in stable monolayers or sub-monolayers (Fig. 1f).

To ensure that the CdSe QDs remained intact and photo-active under the chemical reaction conditions, we performed control experiments in which the QDs were heated at 95 °C either without or with hydrazine but in the absence of GO in the water phase. Digital images and fluorescence spectra of the QDs before and after heating are provided in the Supplementary Information. The 2.9 nm CdSe QDs appear light yellow before heating (Supplementary Fig. S5, left). The color remains after heating with hydrazine at 95 °C (Supplementary Fig. S5, right). The fluorescence spectra recorded before and after the treatment also indicate that CdSe QDs retain the 560 nm peak (Supplementary . S6a and S6b). When the QDs were heated in the presence of hydrazine, no peak shift is observed either but the intensity is reduced, most likely due to slight quenching by hydrazine (Supplementary Fig. S6c). Little effect on the ET kinetics was observed, evidenced by similar kinetic photoluminescence (PL) spectra recorded before and after the treatment (see the discussion in the ultrafast spectroscopic analysis part).

**Figure 2:**
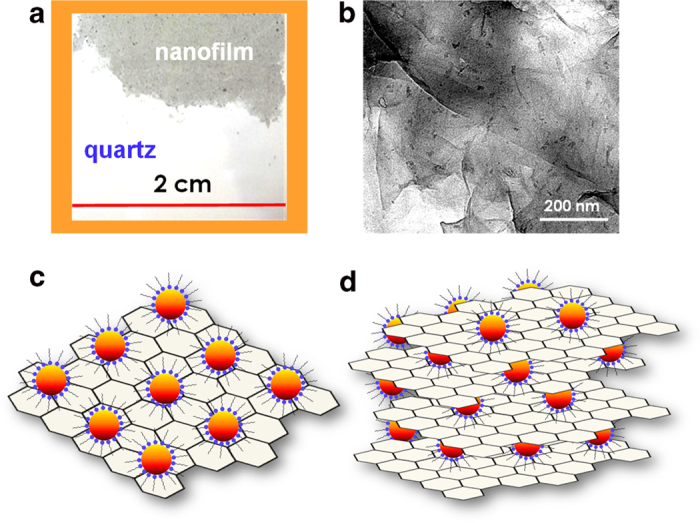
**Morphologic observations of QDs doped RGO nanofilms.** (**a**) Photograph of the nanofilm on a quartz substrate. (**b**) A TEM image of QD-doped RGO nanofilm. (**c**,**d**) Schematic illustrations of the proposed structures for QDs on a single graphene nanosheet (**c**) and of QDs multiply sandwiched in a graphene film (**d**). Not drawn to scale in (**c**) and (**d**).

By pre-inserting a quartz or glass substrate into the beaker and removing the reaction solution, a large-area RGO-QDs nanofilm is formed directly on the substrate surface via spontaneous and fast growth^40^. The RGO-QDs films on the quartz surface exhibit grey to dark brown color, depending on the film thickness which is mainly controlled by the concentration of GO. The color is dominated by graphene as the color of QDs is weak compared with graphene (e.g. Fig. 2a). The presence of QDs in the organic phase thus does not affect spontaneous and fast growth of nanofilms, as long as the appropriate ratio (w/w) of GO to QDs is used.

In the pure RGO nanofilms (i.e. without QDs), the graphene nanosheets are organized via π-π stacking with wrinkles^40^. The nanofilm is composed of multilayered sheets as observed by TEM (Supplementary Fig. S7), and the crystal orientation of RGO nanofilms revealed by HRTEM is (002) (Supplementary Fig. S7c). When meeting with RGO sheets at O/W interfaces, the QDs are attached to the graphene nanosheets via hydrophobic interactions (Fig. 2b and Supplementary Fig. S8). The (111), (220), and (311) crystal orientations were revealed by HRTEM (Supplementary Fig. S8d). On the basis of these measurements, we propose the schematic illustrations of QDs on a single graphene nanosheet in Fig. 2c and of QDs sandwiched multiply in a graphene film in Fig. 2d.

### Structural features of QD-RGO hybrid nanofilms

**Figure 3:**
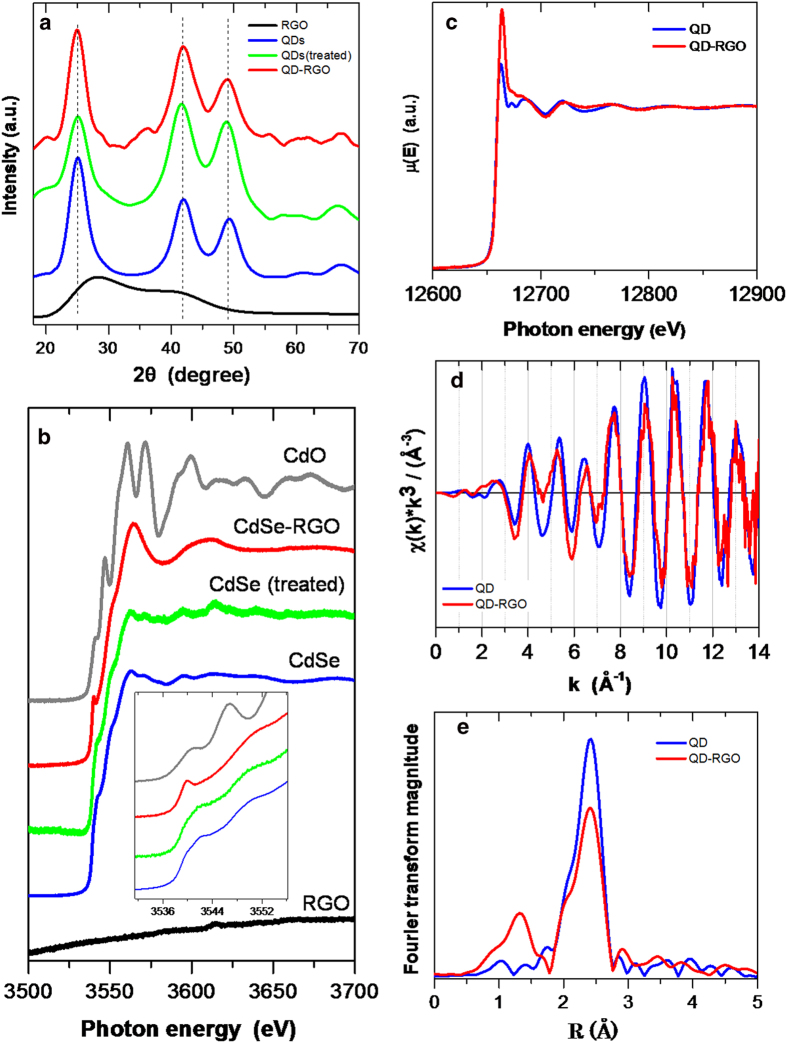
**Structural characterization of various graphene nanofilms by XRD and EXAFS.** (**a**) XRD spectra of various samples. (**b**) Cd XANES L edges of RGO, QDs, QDs (treated), QDs-RGO, and CdO samples. The inset in (**b**) is a zoomed part for highlighting the edge structures. (**c**) Se EXAFS edges of QDs and QDs-RGO samples. (**d**) The k^3^ weighted EXAFS chi (k) functions. (**e**) Fourier transform magnitude of k^3^.chi(**k**) which gives the probability of having a neighbor at a distance R from the Se atom.

Following the synthesis and morphologic characterization of the QD-RGO hybrid films, we studied their structures in detail. The crystalline structures of QD-RGO nanofilms together with the reference systems were *first* analyzed by X-ray diffraction (XRD). This technique offers a general overview of the crystallinity of a sample and the detailed crystalline structure, i.e. symmetry/phase and lattice constants for highly-ordered materials. As shown in Fig. 3a (blue line), the XRD pattern of pure QDs exhibits the typical characteristics of Zinc-blende CdSe structures with space groups of F-43m and a lattice constant of 6.14 Å. Three major peaks are observed at 2θ of 26°, 42°, and 49°, corresponding to the crystal planes (111), (220) and (311)^42^. The slight broadening of all three peaks indicates a size reduction of the QDs compared to bulk CdSe. In contrast, the XRD pattern of the pure RGO nanofilms is broad and featureless (Fig. 3a, black line), attributed to the (002) plane of O/W treated RGO^43^,^44^,^45^. We compare the XRD patterns of pure QDs treated with O/W (green line) and QD-RGO (red line) samples. They both retain the structural characters of pure QDs without any peak shift or broadening. The results suggest that neither O/W treatment nor attachment to RGO causes changes in the QDs lattice structure or in the size distribution of crystallized QDs. These observations are consistent with the HRTEM analysis (Supplementary Fig. S8d). However, the broad and featureless background occasionally observed is likely induced by some amorphous materials that cannot be structurally resolved by XRD. It is thus necessary to complement the characterization of our samples by X-ray absorption spectroscopy (XAS). XAS does not require a material having long range order and can offer more accurate structural information about the first coordination sphere around the absorbing element.

As shown by the Cd XANES L3 edge spectra (Fig. 3b), we can see the exact same absorption features for the QDs (blue trace) and the O/W treated QDs (green trace). This indicates that the local order in the QDs and the O/W treated QDs found in the oil phase of the O/W mixture is the same. This is largely as expected, as the XRD patterns are indeed similar too (Fig. 3a). The noise recorded in the green trace is simply due to the lower density of CdSe QDs in the QD-RGO samples than that in the pure QD samples drop-casted on quartz substrates. It should also be noted that the pure RGO samples do not show any absorption jump, as expected. However, the XANES L3 edge for the QD-RGO samples displays some differences (Fig. 3b, red trace) for some of the sampling spots investigated during the beam time. There is clearly an additional pre-edge absorption peak at 3540 eV in the QD-RGO sample, as shown in the inset of Fig. 3b, along with an overall alteration of the near-edge spectral lineshape. This indicates that another species containing Cd (denoted as Cd-X) is present in the QD-RGO samples. For the species Cd-X, however, the neighboring atoms of the Cd atom are not Se atoms, which is in principle the only possibility in pure QDs. The comparison with the CdO reference (Fig. 3b, grey trace) shows that the Cd-X species is not a simple CdO oxide either. The same trends are observed at the Se K edge in the QD XANES (Fig. 3c). This leads to the conclusion that there is at least another Se-containing species (denoted as Se-Y) in the QD-RGO samples as well.

We *then* turn to the analysis of the extended X-ray absorption fine structure (EXAFS) at the Se K edge to investigate the Se coordination in more detail. First, from the k^3^ weighted EXAFS function in k space, we can see a uniform, strong, well-defined, undamped oscillation for both the pure QDs and QDs-RGO samples until k = 14 Å^−1^ (Fig. 3d). This means that the degree of disorder quantified by the Debye Waller factor is not significant. In other words, the QD-RGO samples probably contain Cd-X and Se-Y species but do not present compositional disorder, i.e. overall retaining well defined first coordination shells after QD/RGO attachment (Fig. 3d.). This feature is even better reflected in the Fourier transform in R space, as shown without phase shift correction (Fig. 3e). The data clearly offer the evidence for the distance at which a neighbor of Se can be found. The main peaks for both pure QD and QD-RGO samples appear around 2.40 Å with a narrow distribution. This distance is the typical Cd-Se atom spacing in CdSe QDs. This peak is found at 2.63 Å after the phase shift correction in real space. However, the intensity of this peak decreases in QD-RGO and an additional peak appears at a shorter distance (1.3 Å). This indicates a coordination of Se with neighbors at a shorter distance (e.g. with low Z neighboring atoms C, N, O, since the distance is shorter than 2 Å).

As for the origin of this additional low-Z coordinated atom, *first* we can exclude the possibility of surface Se-O. Such a bond is present in a 1:4/3:4 ratio in the pure QD, but this peak is not observed in the pure QD samples. *Secondly*, the assignment as originating from Se-C where C belongs to RGO is not warranted either. This is due to the fact that only a very small amount of the surface Se atoms are involved in the bonding, regardless of the bonding nature (covalent, chemisorbed or physically adsorbed). We therefore suggest the following possible origin: during the synthesis, some QDs may not be fully passivated by the capping agent. As a consequence, such QDs react with hydrazine and decompose into Cd and Se ions. The charged ions could be trapped by the dangling bonds of RGO to form isolated Cd-RGO or/and Se-RGO species. In contrast, fully passivated QDs can survive in the oil phase and indirectly attach to RGO nanosheets via the OA alkyl chain while retaining their well-defined nanocrystalline structures. We believe that the majority of the QDs in the QDs-RGO samples are, however, fully protected by OA, as supported by the XRD patterns (Fig. 3a) and the TEM analysis (Supplementary Fig. S8).

### Ultrafast spectroscopy of electron transfer kinetics

**Figure 4:**
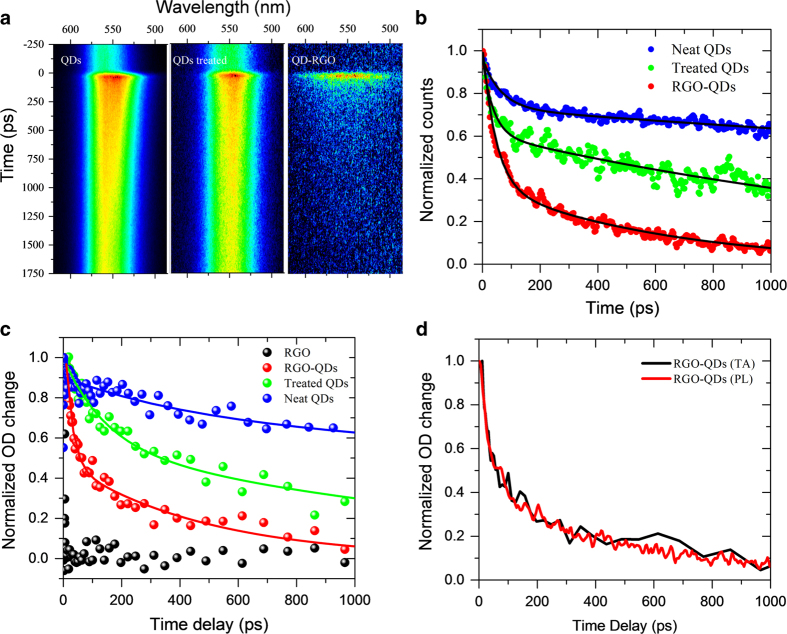
**Ultrafast spectroscopy analysis of excited state dynamics of QDs.** (**a**) Comparison of time-resolved photoluminescence (TRPL) kinetics spectrograms of pure QDs, treated QDs and QD-RGO samples. (**b**) PL decay kinetics extracted from the TRPL spectra shown in (**a**) at the wavelength 560 nm with bi-exponential fits (black lines). (**c**) Ultrafast transient absorption (TA) spectra of three types of samples, where the bold solid lines are the bi-exponential fittings. (**d**) TA and TRPL kinetics of RGO-QDs showing the same decay trace up to 1 ns.

We analyze the photo-induced kinetics of the QD-RGO system using time-resolved photoluminescence (TRPL) and transient absorption (TA) spectroscopies. First, we recorded TRPL spectra to extract the excited state dynamics (Fig. 4a,b). In order to identify different kinetic components, three types of samples were investigated in parallel: (1) pure OA capped QDs (i.e. neat QDs); (2) QDs extracted from the upper oil phase after QD-RGO synthesis (i.e. QDs treated). The upper solution was first centrifuged to remove the residual QD-RGO sheets and then dropcast on the glass (or quartz) substrate. It therefore only contains the neat QDs unattached to RGO. Since such QDs have been treated with heat and hydrazine during the reaction, they constitute a more precise reference system to analyze the excited state dynamics of QD-RGO sample than neat QDs. (3) QD-RGO extracted from the O/W interface (QD-RGO). The PL spectra show that three types of samples have the same emission band at 560 nm (Fig. 4a).

Compared with untreated and treated pure QD samples, the lifetime of RGO-QDs is drastically shortened, demonstrating efficient quenching by RGO. We used a bi-exponetial function: A_1_ exp(–t/τ_1_)+ A_2_ exp(–t/τ_2_) to fit the PL decay kinetics at the wavelength of emssion maximum (560 nm) (solid lines in Fig. 4b). It should be noted that during the measurements the excitation flux was kept very low (2.2 × 10^10^ photons/cm^2^/pulse) giving an average number of excitons per QD (<*N>*) 2 × 10^−5^. The Auger recombination of multiexcitions is negligible at such low excitation flux^46^,^47^. As summarized in Table 1, the PL decay of pure OA capped QDs exhibits a major long-lived component (7.3 ns) which can be attributed to radiative recombination in the QDs^18^. The fast component (65 ps) represents the surface trapping, especially hole trapping^46^. When the QDs were treated with hydrazine, the emission lifetime is shortened compared to untreated (or pure) QDs. Given our observation of partial decomposition of some QDs by hydrazine as evidenced by the XAS characterization, we conclude that the shortening is caused by the creation of additional surface defects or dangling bonds serving as trapping centers for electrons or holes. The PL decay in RGO-QDs sample was drastically shorter than in the other two samples. We have calculated the average PL lifetime <τ> as:


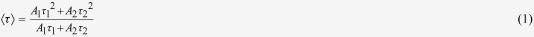


where A_1_ and A_2_ are the amplitudes of the two exponential components. As summarized in Table 1, the average PL lifetime of RGO-QDs sample <τ>_RGO-QDs_ (556 ps) is much shorter than that of the treated QDs <τ>_RGO-QDs_ (2853 ps). As the two samples experience the same hydrazine treatment in the same batch of solution, this extra quenching process can be attributed to the new exciton depopulation pathway through the graphene sheet. The rate constant of such depopulation process (*k*) can be calculated as:


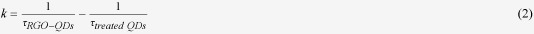


Here <τ>_RGO-QDs_ and <τ>_treated QDs_ represent average lifetimes of RGO-QDs and treated QDs, respectively. We obtain the rate constant *k = *1.3 × 10^9^ s^−1^. To evaluate the reproducibility of measurements, we did measure at least three different batches of samples prepared with the same experimental condition in parallel. The deviation of quenching rates is typically within 7% (e.g. Supplementary Fig. S9).

In general both energy transfer and charge separation can account for PL quenching. The Dexter type energy transfer can occur in case of wave function overlap between the donor and acceptor^48^. Due to the capping OA molecules such overlap is negligible here. The Förster resonance energy transfer (FRET) has been reported in several other QD-graphene composite systems^36^,^49^,^50^. In our system, however, the maximum FRET rate from QDs to graphene can be calculated as:^51^


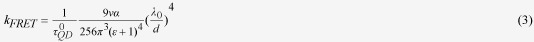


where α is the fine-structure constant of graphene (the absorbance for monolayer graphene is given by πα ≈23%), d is the QD to graphene distance, τ^0^_QD_ is the lifetime of neat QDs; ν = 1 (ν = 2) when the emitter dipole is oriented parallel (perpendicular) to the graphene, ε is the dielectric permittivity of the medium, and λ_0_ is the emission wavelength of the emitter (QDs). Given the distance (d) between the QD center to graphene sheets 3.5 nm (i.e. the QD radius (1.5 nm) plus the 2 nm chain length of oleic acid^52^), τ^0^_QD_ = 7.3 ns, α = 1/137, ε = 4.5 for glass substrate, emission wavelength of QDs λ_0_ = 560 nm, and randomly oriented dipole constant ν = 4/3 (ν = (2/3)ν_||_+(1/3)ν_⊥_), the maximum FRET rate in our system is estimated to be 3.9 × 10^8^ s^−1^. This estimated rate is significantly lower than the measured quenching rate (1.3 × 10^9^ s^−1^), suggesting that the FRET does occur but is a minor process in the present system. We therefore conclude that charge transfer is a dominating photo-induced process in the QD-RGO nanocomposite.

We further identified the charge separation to be electron injection from the conduction band (CB) of QDs to RGO by a comparison of TRPL spectra with TA analysis. The density of the excited electron states at the band-edge transition of CdSe QDs is significantly smaller than the corresponding density of the hole states^46^. In the effective-mass approximation, the lowest excited electron state is only twofold degenerate^53^. In addition, the hole states are more closely spaced in QDs. As a consequence, the signal of excited state bleaching of CdSe QDs is dominated by the state filling of electrons rather than the holes. At the same time the PL is sensitive to both carriers (i.e. electrons and holes). Consequently, the TA signal provides a means to ascertain or preclude the involvement of electron depopulation in PL quenching^46^. As shown in Fig. 4c, the TA kinetics probed at the maximum of bleaching (560 nm) of neat QDs and treated QDs exhibits similar average lifetime as TRPL. In particular, the normalized TA and TRPL decay curves of RGO-QDs sample almost follow each other (Fig. 4d). This comparison offers strong evidence that the emission quenching is caused by the depopulation of excited electrons in QDs.

An interesting concern is the effect of QD sizes on charge or energy transfer dynamics. We used a simplified way to evaluate this issue, i.e. by monitoring the dynamics of PL with different wavelength, as it reflects the size distribution in our QDs. We picked PL dynamics of RGO-QDs at two different wavelengths (500 nm and 600 nm) corresponding to a mean size of 2.3 nm and 4.6 nm, respectively. The PL decay follows the almost same trend (Supplementary Fig. S10), indicating that the quenching rate is almost independent on the QDs size in our case. Although the reason for that is not clear yet, the results can simplify our conclusion that the electron transfer rate depends mainly on the QD-graphene coupling.

### Rational analysis of the nature of charge transfer

**Figure 5:**
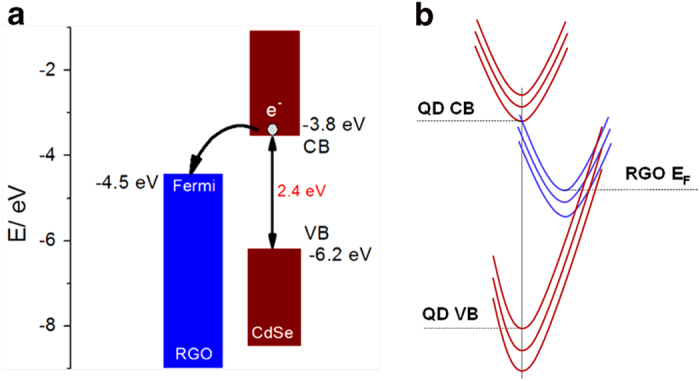
Band energy diagram and potential free energy surface representation of QD-graphene composites . (**a**) Energy level alignment of QD and graphene. CdSe QDs with a size of 2.9 nm exhibits a bandgap of 2.4 eV^45^. (**b**) Free energy representation of the QD-graphene system.

The band energy diagrams shown in Fig. 5 prompt theoretical observations regarding the prevalence of electron or hole transfer. Electron transfer involves, *first* transfer of photo-excited electrons from the bottom of the QD conduction band (CB) to the graphene Fermi level or levels above this level, in a weakly exothermic process, i.e. the energy gap is ≤ 0.7 eV (Fig. 5a). Hole transfer, or electron transfer is in the opposite direction, i.e. from electronic levels around the graphene Fermi level to levels below the top of the QD valence band (VB), liberated by QD photo-excitation. The energy gap of this process is much larger, namely 1.7 vs 0.7 eV. *Secondly*, electron or hole transfer between the molecular-size QD (2-3 nm) and graphene in the condensed matter environment (here solid state) must involve substantial nuclear reorganization, say low-frequency collective vibrational reorganization in the graphene matrix. The potential (free) energy representation shown in Fig. 5b therefore also applies. The values of the thermodynamic (free) energy (driving force) vs. the dynamic environmental reorganization free energy are here crucial determinants.

We have no precise estimates for the QD/graphene reorganization free energy, but in the apolar graphene matrix this quantity is unlikely to exceed the energy gap in the QD/graphene electron transfer process. As no vacant electronic acceptor levels in the QD band gap are available, hole transfer from QD VB to graphene or equivalently electron transfer from graphene to QD VB therefore involves an energy gap that exceeds significantly the environmental nuclear reorganization free energy. In other words, hole transfer belongs to the strongly exothermic inverted reaction free energy region. In this region the rate constant drops approximately exponentially with increasing energy gap as first introduced for multi-phonon solid state electronic relaxation processes by Pekar^54^ and by Kubo and Toyozawa^55^, and broadly known for non-radiative electronic relaxation in large molecules^56^. In contrast, electron transfer belongs to the “normal” free energy region, where the charge transfer rate is much larger and increases with increasing energy gap. We therefore conclude that the QD/graphene fluorescence decay processes are by far dominated by electron transfer rather than hole transfer.

Efficient electron transfer in QD-RGO composites with long D-A spacing (>1 nm) has not been reported before, although long-range charge transfer in protein systems non-covalently attached to electrodes is well documented^57^,^58^. In previous studies of QD-RGO composites using oleic acid as linker molecule, only slow energy transfer with a rate constant of 1 × 10^8^ s^−1^ was identified^49^. Charge transfer from QDs capped with the short linker molecule pyridine to single graphene nanosheet was recently studied^37^. Although oleic acid is 4 times longer than pyridine, the rate does not decay drastically considering the exponential distance dependence of the transfer rate from donor to acceptor^59^. This surprising result can be explained by the fact that the QD-RGO composite formed by interfacial self-assembly creates multiply sandwiched stacking between the QDs and RGO (Fig. 2d). Such structure opens multiple channels for electron transfer leading to enhanced total transfer rate. Similar effect has been noted before in few charge-transfer systems such as a fullerene–ferrocene dyad linked by a rigid bilinkage^60^.

### Photocurrent generation in liquid-junction QD-sensitized solar cells

**Figure 6:**
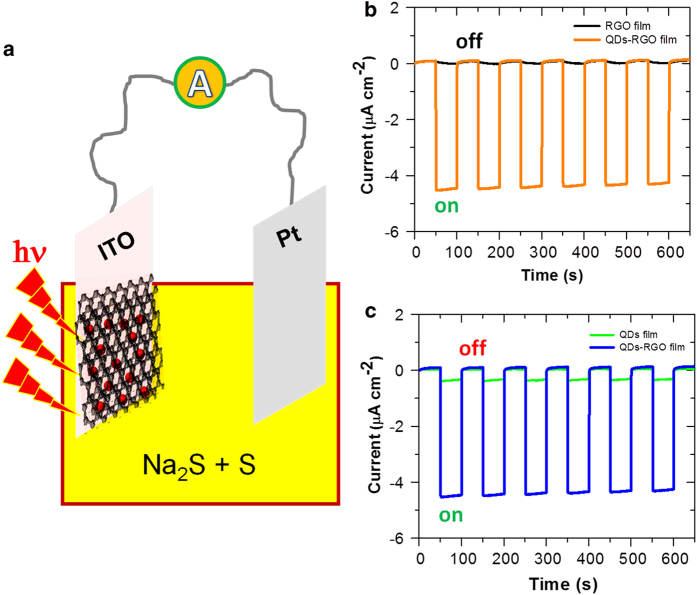
Photocurrent generation of QDs-RGO nanofilms in a liquid-junction QD-sensitized solar cell . (**a**) A schematic illustration of photoelectrochemical setup in an aqueous solution containing 1 M Na_2_S and 1 M S electrolytes saturated with argon. ITO with QDs-RGO film and Pt wire were used as working and counter electrodes, respectively. (**b**) Comparison of photocurrent responses of QDs-RGO and pure RGO films. (**c**) Comparison of photocurrent responses from QDs-RGO and pure QD films. The current-time curves were recorded at open circuit potentials with white light used as a simulated solar light source for the photocurrent generation (input power 100 mW cm^−2^ and λ >  400 nm).

In order to further verify the conclusion of electron transfer over energy transfer, we performed photoelectrochemical measurments. Figure 6a shows a schematic illustration of an in-house designed photoelectrochemical cell. The QD-RGO nanofilm grown on an ITO electrode was used as a photoanode, with pure RGO or treated QDs modified ITO electrodes for the control experiments. Na_2_S and S were used as electrolyte and redox species. A mimic solar light source was used to illuminate the working electrodes. Pure RGO films generate negligible photocurrent (black lines in Fig. 6b), and treated QD films only give rise to a small photocurrent response (green lines in Fig. 6c). In contrast, QD-RGO nanofilms show a fast response (within seconds) to light illumination with a remarkably enhanced photocurrent generation (Fig. 6c). Compared to the pure QD films, photocurrent generation is enhanced by 15-20 times with sandwiched confinement of QDs in the RGO matrix. Moreover, the On-Off photocurrent switching to white light is completely reversible (Fig. 6b,c). The large photocurrent generation is a clear indication that directed fast movement of charges within RGO-QDs composite must occur, which in turn means that charge separation indeed takes place after photoexcitation.

To further prove sandwiched confinement of QDs in the graphene matrix (as proposed in Fig. 2d) rather than just adsorption on the surfaces of RGO film, we prepared an additional type of reference samples. We *first* prepared a pure RGO film on ITO by the O/W reduction (Supplementary Fig. S3), and we *then* immersed RGO-ITO electrodes in QDs-containing toluene solution for the adsorption of QDs on the RGO film surface. The resulting electrodes were used in the photoelectrochemical measurements under the same conditions as described above. Clearly, such systems can only generate approximately 30% of the photocurrent current that is generated by the RGO-QDs integrated composites (Supplementary Fig. S11).

The observed conspicuous photocurrent relates to the prevalence of *electron* transfer rather than *hole* transfer from the CB of photo-excited QDs to graphene (Fig. 5). Photo-excitation generates virtually exclusively electrons in the graphene matrix. The most likely scenario is then that the electrons are transferred through the metallic leads to the Pt-counter electrode where S is reduced to S^2−^. The holes left in the QDs are filled by oxidation of S^2−^ to S. The ITO/graphene/QD electrode is thus the anode and the electrochemical process dominated by *electron* injection from this electrode to the Pt-cathode. In short, the photoelectrochemical experiments demonstrate: 1) QD-graphene hybrid nanofilm is a highly active optoelectronic material, which can be directly used as a photoanode in liquid-junction QD-sensitized solar cells; and 2) the results are consistent with those obtained by the ultrafast spectroscopy and rational analysis, i.e. electron transfer is a pre-dominant process in the present system.

## Discussion

Large-area QD containing thin films are highly desirable for the fabrication of QD-sensitized solar cells and other QD based optoelectronic devices. Furthermore, it is noted that liquid-junction solar cells are on the way to become a new-generation efficient energy conversion setup for utilization of solar energy. To this end, graphene offers both an efficient scaffold platform and an electronically functional component. The present study has shown the feasibility of such type of combinations. The two-phase solution processing facilitates the formation of QD doped RGO nanofilms where the QDs retain their crystalline structures. We have employed ultrafast spectroscopies to study charge transfer kinetics in detail and to identify the nature of charge transfer, which is accompanied by rational analysis and photoelectrochemical measurements. A comparison of PL and TA spectra leads to the conclusion that electron injection rather than hole injection occurs from photoexcited QDs to graphene. The photoelectrochemical results offer strong evidence that electron transfer is a pre-dominant process over Förster energy transfer, in contrast to most previous reports. In short, graphene nanosheets enable multiple sandwich-like confinement of the QDs with a dramatic improvement of electronic coupling, leading to fast long-range electron transfer from QDs to graphene and a 15-20 fold enhancement in photocurrent generation. The performance of hybrid films, including electron transfer rate and photocurrent generation, could be further improved by removing the capping ligand oleic acids from QD surfaces, as suggested by a recent report^38^. However, the stability of hybrid films could then become an issue of concern.

It is argued that electron transfer, rather than hole transfer is mostly likely to prevail in a charge transfer mechanism and that electron transfer belongs to the activationless free energy region, while hole transfer belongs to the “inverted” free energy region. The simplest representation of the rate constant, k_ET_ is then


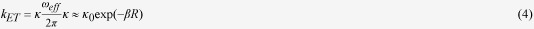


with no activation free energy term. ω_eff_ is the effective vibrational frequency of all nuclear modes reorganized, and κ the electronic transmission coefficient which can be given the approximate exponential distance dependence form shown. R is the ET distance and κ_0_ a quantity of the order unity. β (length^−1^) is the decay factor often with β ≈ 1 Å^−1^ for ET through covalently bonded molecular matter but with both smaller and larger values reported.

We are unable to directly calculate the rate constants with sufficient confidence since the structural details of the capping oleic acid relative to QD and the graphene layers are not known. What we can do in comparing this simple formalism with the fluorescence decay and transient absorption date is to estimate the ET parameters needed for accordance with the data. The maximum value of ω_eff_ would be ≈ 10^13^ s^−1^ (10^14^ s^−1^), giving for the observed rate constant of 1.3 × 10^9^ s^−1^ a transmission coefficient of κ ≈ 10^−4^-10^−5^ for ET to be competitive. This corresponds to a β-value of about 0.5 Å^−1^ if R is 20 Å (the oleic acid chain length) and 0.3 Å^−1^ if R is 35 Å (the QD also included.). These values appear notably smaller compared with the often recorded value of β ≈ 1 Å^−1^ but two observations are appropriate.

One observation is that the β-value depends sensitively on the tilt angle of the molecular linker giving a smaller value for larger tilt angles. This effect is rooted in the nature of the dominating molecular orbitals in the superexchange pathway between the donor (here the QD) and the acceptor (graphene). These orbitals change as the tilt angle changes. The other observation is that the process presently considered involves an excited electronic state whereas β-values around 1 Å^–1^ determined from electrochemical processes at self-assembled molecular monolayers involve electronic ground states. This could lower the β-value as the energy gap between the donor and the intermediate molecular electronic levels, Δ, is significantly smaller, cf. the equation^56^


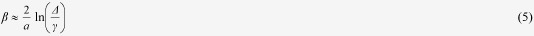


where *a* is the average extension of an intermediate molecular fragment and γ the electronic coupling between two nearest neighbor fragments in the molecular conduction chain.

High-energy excited states and low β-values finally raise the question whether electron “hopping”, say via the orbitals associated with the oleic acid double bond may be a contributing channel. This would further lower the apparent β-values. Weak distance dependence thus cannot distinguish between exponential and other well behaved distance dependence such as the inverse polynomial dependence characteristic for hopping. The conclusion is that the extracted ET parameters can well be in ranges where the ET decay channel prevails. A definite proof requires, however, more detailed structural information about the RGO-QD composites than presently available.

The present work is focused on the 2.9 nm CdSe QDs capped with oleic acid. However, the method applies to different-size CdSe QDs as well as to other types of QDs. CdSe QDs with other sizes were synthesized for preparation of RGO-CdSe QDs films (Supplementary Fig. S12), in order to address size-dependent effects in the enhancement of photoinduced excited-state kinetics in on-going research. For example, 2.4-3.1 nmQDs were synthesized, giving rise to absorption bands at 505, 526, 542 and 551 nm with distinct colors (Supplementary Fig. S12a). RGO-CdSe QDs samples were also prepared using O/W induced interfacial assembly (Supplementary Fig. S12b). In addition, CdSe QDs capped with different types of ligands instead of oleic acid have been used for the formation of RGO-CdSe QDs thin films, although they need to be studied in detail.

## Methods

### Chemicals and Materials

Graphite power (<20 μm, synthetic), potassium permanganate (KMnO_4_, ≥99%), potassium persulfate (K_2_S_2_O_8_, ≥99%), potassium dihydrogen phosphate (K_2_HPO_4_, ≥99.999%), phosphorus pentoxide (P_2_O_5_, ≥98%), sulfuric acid (H_2_SO_4_, 95-97%), hydrazine hydrate solution (50-60%), ammonia solution (25%), toluene (99.9%), Cadmium oxide (CdO, 99.5%), selenium (Se,>99.5%), oleic acid (OA,>99%), octadecane (ODE, ≥95%), trioctylphospine (TOP,>90%), Sodium sulfide (Na_2_S) and Sulfur (S, ≥99.5%) were all from Sigma-Aldrich and used as received. Milli-Q water (18.2 MΩ cm) was used throughout.

### Preparation of graphene oxide

Graphene oxide (GO) was prepared according to our previous procedure^40^,^41^, based on the modified Hummer’s method^61^. The detailed procedure is described as follows. The whole procedure for the preparation of graphene oxide (GO) included two steps. In the first step, pre-oxidized graphite was prepared first. Graphite powder (5.0 g) was slowly added with strong stirring for 3 h into a hot water bath (80 °C), which contained concentrated H_2_SO_4_ solution (7.5 ml), P_2_O_5_ (2.5 g) and K_2_S_2_O_8_ (2.5 g). After cooling to room temperature, the mixture was diluted with Milli-Q water, filtered and washed with Milli-Q water until the waste solution reached neutral pH. In the second step, pre-oxidized graphite powder (1.0 g) was slowly added into concentrated H_2_SO_4_ solution (23 ml) in an ice-water bath (0 °C) environment. KMnO_4_ (3.0 g) was then added to the mixture under slow stirring. The mixture was kept at 35 °C for about 2 h with slow stirring. 30% H_2_O_2_ solution and Milli-Q water were added next into the mixture. Depending on the concentration wanted, the color changed from dark green to bright yellow. Finally, the mixture was washed with HCl solution to remove residual metal ions. The raw GO was then centrifuged and dialysed several times to obtain highly purified and dispersed GO.

### Preparation of CdSe quantum dots (QDs)

The oleic acid (OA) protected CdSe QDs were synthesized using our previous procedure^16^,^18^,^22^,^25^, where different sizes of CdSe QDs (2.4 nm, 2.7 nm, 2.9 nm, 3.1 nm) were prepared, corresponding to absorption bands at 505, 526, 542, 551 nm, respectively. In brief, 10.5 g trioctylphosphine (TOP) and 3 mmol Se powder were dissolved in 10 ml octadecane (ODE) for 1 h stirring to receive a TOP-Se solution. At the same time, under the protection of N_2_ atmosphere of a three-neck flask, 5.54 g OA and 0.51 g CdO were slowly added into 70 ml ODE solution, and then heated to 180 °C forming a clear solution. The first TOP-Se solution obtained was quickly transferred into the three-neck flask. During the injection of TOP-Se solution, the Cd^2+^ precursor solution was heated to 180, 200, 220, and 240 °C separately to obtain different sizes of QDs. After about 2 minutes’ reaction, the flask was quickly removed from the hotplate and cooled to room temperature. Methanol and acetone were used to rinse the precipitate of QDs, which was re-dissolved in the toluene for further use.

### Preparation of pure RGO nanofilms by O/W two-phase solution processing

RGO nanofilm was prepared by chemical reduction of GO with hydrazine in the O/W two-phase system^41^. Briefly, mixing GO (0.15 mg ml^−1^) in water solution and toluene together in volume ratio of 1:1, and then separating the two parts in a beaker, leaves toluene as the top layer. 100 μL hydrazine hydrate solution (5%) was added to the mixed solution and left for incubation in a water bath at 95 °C with strong stirring. The reduction reaction was fast and completed within 15 min, but was normally left for 30 min. The graphene nanosheets were spontaneously self-assembled at the interfaces between water and toluene or between toluene and glass wall. No RGO was left in the obviously fully transparent water phase. The as-prepared graphene nanosheets could be transferred onto a special substrate such as glass or quartz for device or other application. Pre-inserting a quartz substrate into the beaker and drop-casting the reaction solution immediately lead to spontaneous and fast growth of large-area RGO nanofilms.

### Preparation of RGO-QDs nanofilms by O/W two-phase solution processing

OA-capped CdSe QDs were dispersed in toluene, which could be used directly in the O/W two-phase system instead of only toluene. These two phases were prepared for synthesizing large-area graphene-QDs nanosheets by the O/W interface method. We attempted different ratios of GO to QDs to prepare the hybrid films. It was found that the optimal ratio is towards a volume ratio of 1:1 with a GO (0.15 mg ml^−1^) water solution and a QDs toluene (8.13 μmol l^−1^) solution. This ratio ensured all individual RGO nanosheets to be decorated with QDs before their self-assembly into nanofilms. Before reaction, CdSe QDs with the orange color are located only in the toluene phase (with 542 nm peak absorption as an example). The dark yellow color in the bottom layer is from GO. After reaction with hydrazine at 95 °C for 30 min, the water phase became clear and colorless, while the QD phase changed to dark color forming RGO doped with CdSe QDs. When carefully examined the QD phase showed that most of the RGO-QDs layer adhered to the interface between toluene and water, and some on the glass wall.

### Photoelectrochemical measurements

Photoelectrochemistry was carried out using an in-house designed electrochemical cell^62^. QDs-RGO, QDs and RGO films were freshly prepared on ITO electrodes (1 × 1 cm^−2^), followed by annealing at 60 °C in an oven for one hour for complete drying. These nanofilm modified ITO electrodes were used as photoanode (or working electrodes), and a Pt plate was used as a counter electrode in the 1M Na_2_S and 1M S electrolyte water solution saturated with Ar. An ORLED RL18 (100 mW cm^−2^) equipped with a UV cut filter (λ > 400 nm) was used to provide simulated solar light for photocurrent response experiments. The photocurrent-time curves were recorded using an electrochemical workstation (CHI 760, USA).

### Spectrophotometry, AFM, TEM and fluorescence

UV-Vis spectra were recorded using an Agilent Instrument Exchange Service Model G1103A. A 5500 AFM System (Agilent Technologies) was used for AFM imaging. All images were acquired in the tapping mode. TEM imaging was performed using a Tecnai G2 T20 (FEI Company, Oregon, USA). Fluorescence spectra were recorded using a (Spex 1681) standard spectrophotometer excited at the wavelength 500 nm.

### X-ray diffraction

XRD measurements were carried out by the Beamline I711 at the MAX IV Laboratory in Lund, Sweden. The incident X-ray wavelength used in these experiments was 0.99 Å. In the sample preparation, 0.5 mm capillaries with colloidal QDs and RGO-QDs solution were gently heated to fully evaporate the solvent in an oven. The XRD patterns were measured at room temperature under ambient atmosphere.

### X-ray absorption spectroscopy

X-ray Absorption Spectroscopy measurements (XANES and EXFAS) were performed using the Beamline I811 at the MAX IV Laboratory, Lund, Sweden. The samples were deposited onto polyimide film. The absorption edges considered in these experiments were Cd L3 and Se K. The measurements were recorded in total X-ray fluorescence yield mode.

### Transient absorption kinetic spectroscopy

Details of the experimental setup are described elsewhere^23^. Briefly, pulses (1.2 mJ pulse energy, 1 kHz repetition rate, center wavelength at 775 nm and pulse duration about 150 fs) generated from the amplified laser (CPA 2001) were split using a beam splitter to generate the pump as well as the probe beams. The second harmonic at 387.5 nm of one of the beams was used as the pump beam. The probe beam at 555 nm was generated using a non-collinear optical parametric amplifier (NOPA). The diameter of the pump beam at the sample was about 5 mm (not focused), while a 50 cm focal length concave mirror was used to focus the probe beam onto the sample. The focus spot size of the probe at the sample was about 0.5 mm and centered at the maximum of the pump beam in order to minimize the effects of intensity variation along the pump beam cross-section on the dynamics^63^. The photon flux of the pump beam on the sample was about 9 × 10^10^ photons per cm^2^ per pulse (average power of 6 μW).

### Time-resolved photoluminescence (TRPL)

The Titanium-Sapphire (Ti-Sp) passively mode-locked femtosecond (fs) laser (Spectra-Physics, Tsunami) was used as the light source for the time-resolved photoluminescence measurments, emitting at 820 nm with 80 MHz repetition rate and 150 fs pulse length. The pulses were then sent to a pulse picker with a single-pass standing-wave acousto-optic crystal, which divided the laser repetition frequency to 8 MHz. A harmonic generator (Photop technologies, Tripler TP-2000B) controlled the laser pulses at the fixed frequency, frequency-doubled to 410 nm. The excitation photon flux was 2.2 × 10^10^ photons/cm^2^/pulse corresponding to <N> about 2 × 10^−5^. TRPL spectra were recorded by a picosecond streak camera (C6860, Hamamatsu) operating in the single-shot mode coupled to a Chromex spectrograph, triggered by a Ti-Sapphire laser. A long-pass wavelength filter from 490 nm was in use in front of the spectrograph in order to cut off the scattering from the excitation pulses.

## Additional Information

**How to cite this article**: Zhu, N. *et al*. Sandwiched confinement of quantum dots in graphene matrix for efficient electron transfer and photocurrent production. *Sci. Rep.* **5,** 9860; doi: 10.1038/srep09860 (2015).

## Supplementary Material

Supplementary Information

## Figures and Tables

**Table 1:** Summary of the key kinetic parameters obtained from bi-exponential fittings to the measured PL decay and TA kinetics spectra.

**Samples**	**A**_**1**_	**τ**_**1**_ **(ps)**	**A**_**2**_	**τ**_**2**_ **(ps)**	**<τ> (ps)**
**QDs (PL)**	0.24	65±5	0.76	7287±185	7266
**QD treated (PL)**	0.39	86±7	0.61	3006±124	2953
**RGO-QDs (PL)**	0.63	47±3	0.37	622±11	556
**QDs (TA)**	0.24	446±108	0.76	7615±665	7484
**QD treated (TA)**	0.43	143±29	0.57	1559±202	1467
**RGO-QDs (TA)**	0.61	24±4	0.39	478±56	445
