# Supplementary material for: Sandwiched confinement of quantum dots in graphene matrix for efficient electron transfer and photocurrent production
Source: Sci Rep. 2015 May 21;5:9860. doi: 10.1038/srep09860 (PMC4649995; doi:10.1038/srep09860)
Supplement: Supplementary Information [file srep09860-s1.pdf]

Supplementary Information for

Sandwiched confinement of quantum dots in graphene matrix for efficient electron transfer and photocurrent production

Nan Zhu[1,||], Kaibo Zheng[2,||], Khadga J. Karki[2], Mohamed Abdellah[2], Qiushi Zhu[3], Stefan Carlson[3], Dörthe Haase[3], Karel Žídek[2], Jens Ulstrup[1], Sophie E. Canton[3], Tõnu Pullerits[2] & Qijin Chi[1]

[1]*Department of Chemistry, Technical University of Denmark, Kemitorvet Building 207, DK-2800 Kongens Lyngby, Denmark.*

[2]*Department of Chemical Physics, Lund University, Box 124, 22100, Lund, Sweden.*

[3]*The MAX IV Laboratory, Lund University, Box 124, 22100, Lund, Sweden.*

[||] These authors contributed equally to this work.

Correspondence and requests for materials should be addressed to Q.C (email: cq@kemi.dtu.dk) or to T. P. (email: Tonu.Pullerits@chemphys.lu.se).

Contents

S1 Synthesis and characterization of GO and RGO nanosheets……………………..………2

S2 Structure of QDs……………………………………………………………………5

S3 Treatment and effects of QDs ………………………………………………………...6

S4 TEM images of hybrid nanofilms……………………………………………………..8

S5 Time-resolved photoluminescence spectra …………………………………………...10

S6 Photocurrent responses of various films…..…………………………………………..12

S7 Photographs of QDs samples with different sizes ……………………………………13

Supplementary Data

S1. Synthesis and characterization of GO and RGO nanosheets

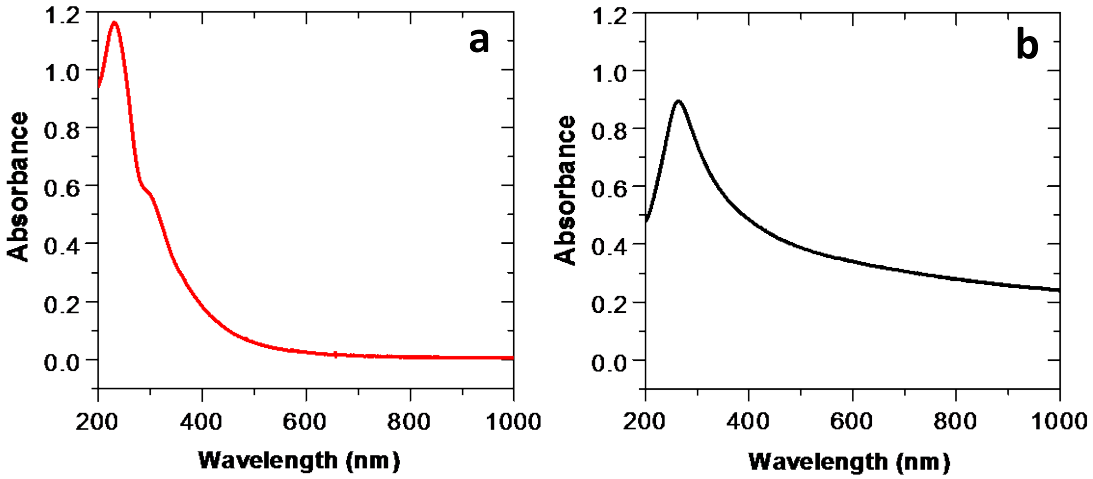

Supplementary Figure S1. Comparison of UV-vis spectra of GO (a) and RGO (b) suspensions.

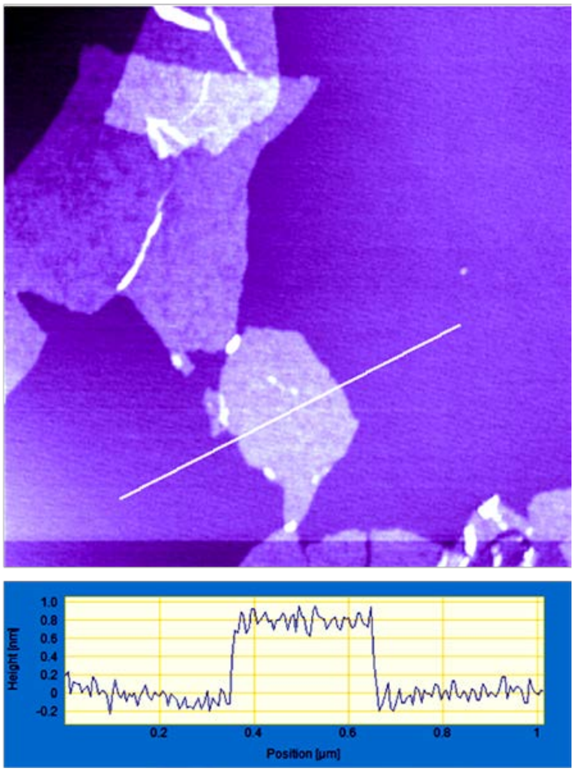

Supplementary Figure S2. AFM image of RGO nanosheets (top) and the cross-sectional profile (bottom). The scanned area: 1.5 µm x 1.5 µm.

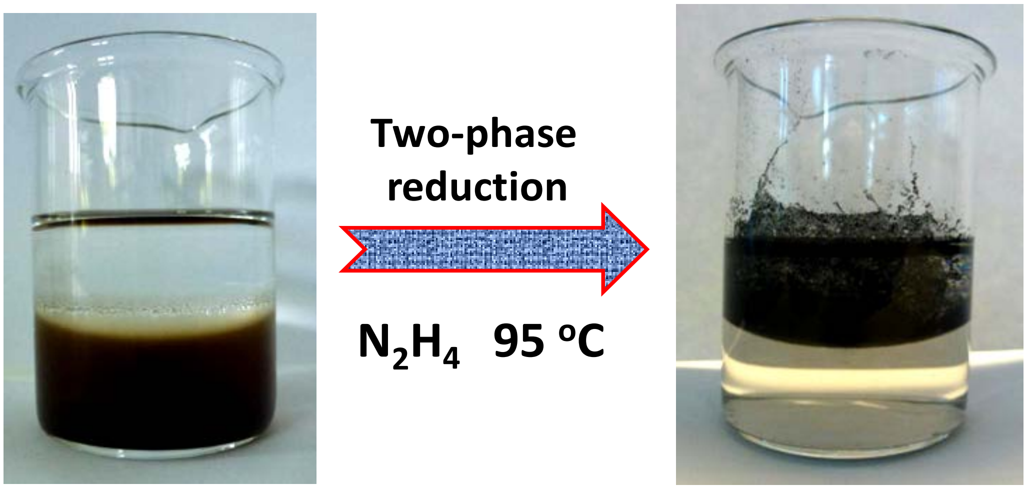

Supplementary Figure S3. Photographs of the samples illustrating two-phase reduction of GO (left, before chemical reaction) to RGO (right, after chemical reaction). The GO concentration is 0.5 mg/ml.

S2. Schematic illustration of the structures of CdSe QDs

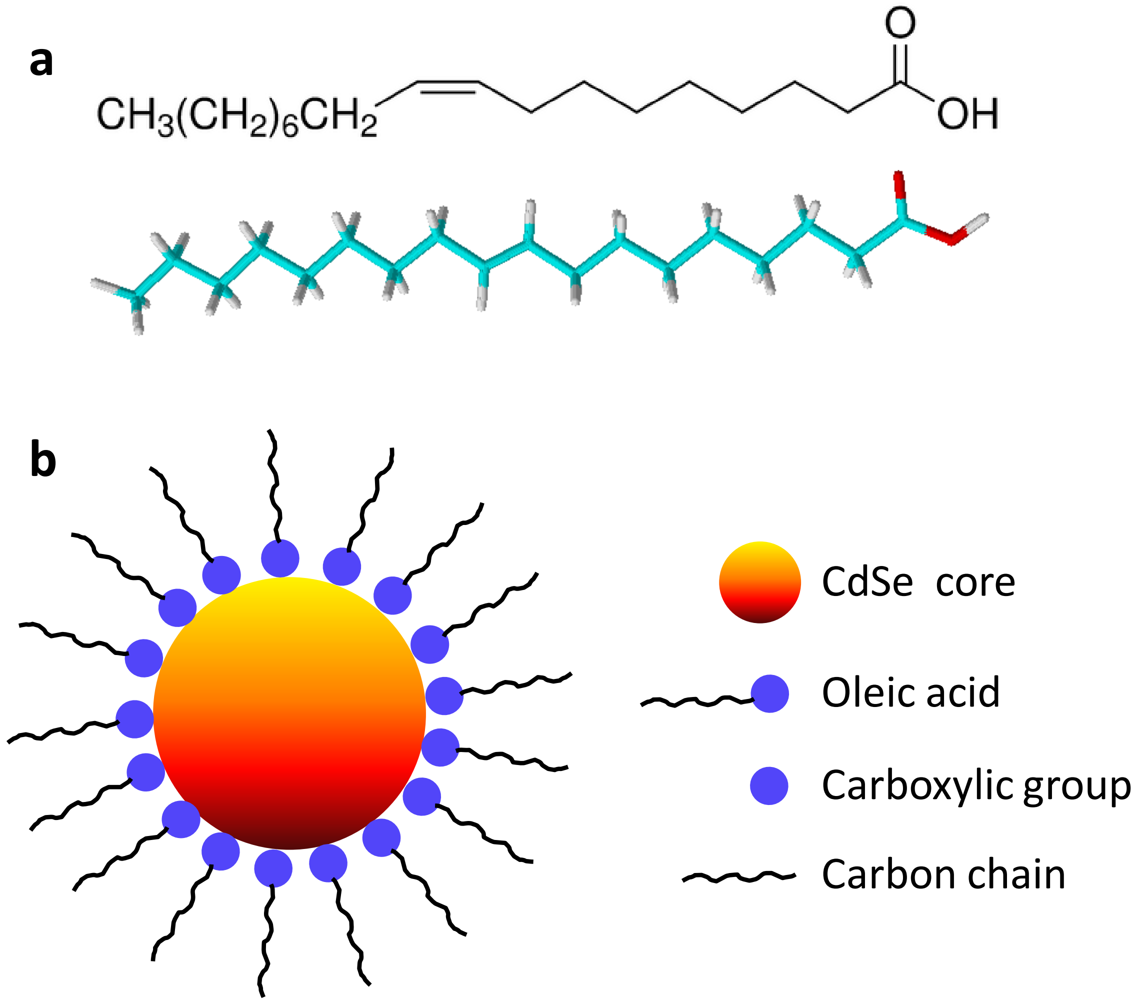

Supplementary Figure S4. (a) Chemical structure of capping ligand, oleic acid (OA); and (b) schematic illustration of OA capped CdSe QD.

S3. Treatment and effects of CdSe QDs

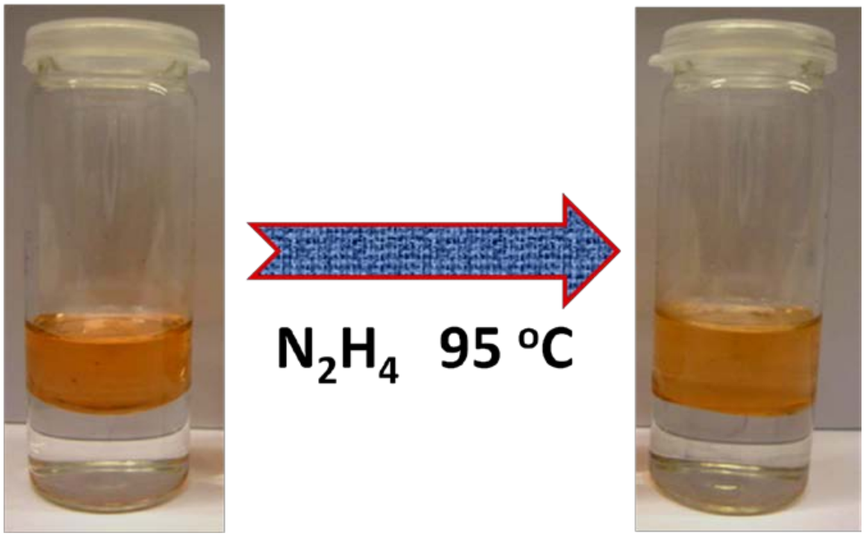

Supplementary Figure S5. Photographs of the QD samples treated under similar experimental conditions.

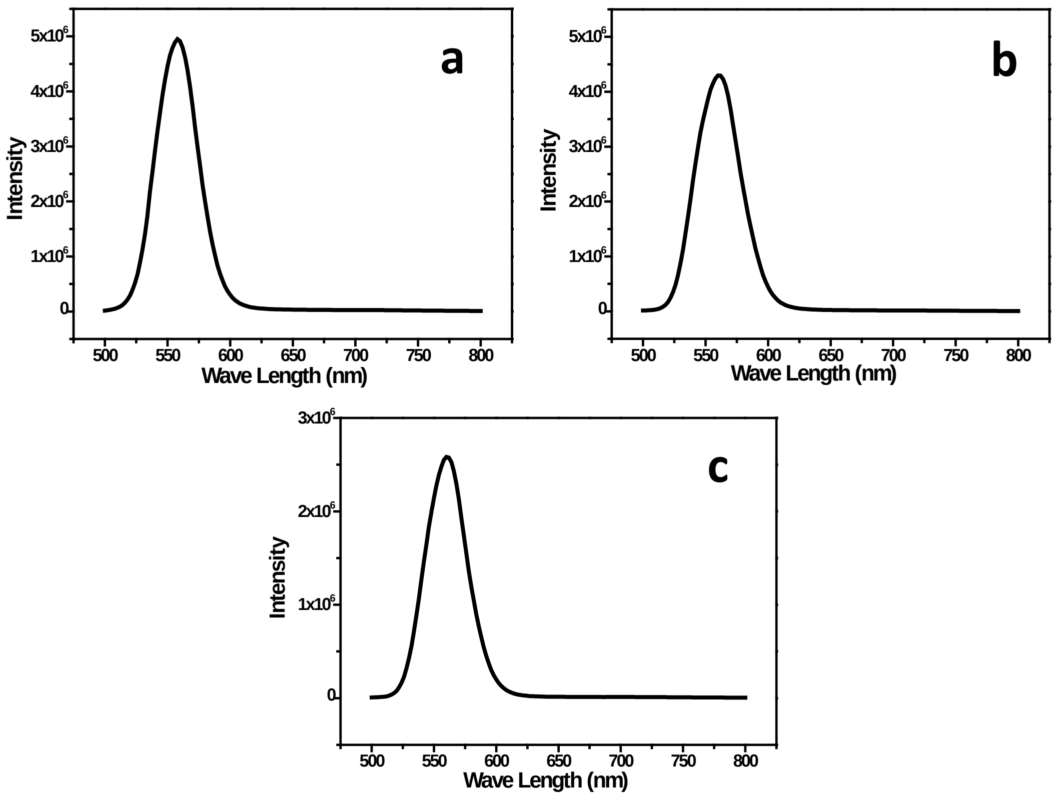

Supplementary Figure S6. Fluorescence spectra of 2.9 nm CdSe dots without treatment (a), with heating treatment only (b) and with heating in the presence of hydrazine (c).

S4. TEM images of various nanofilms

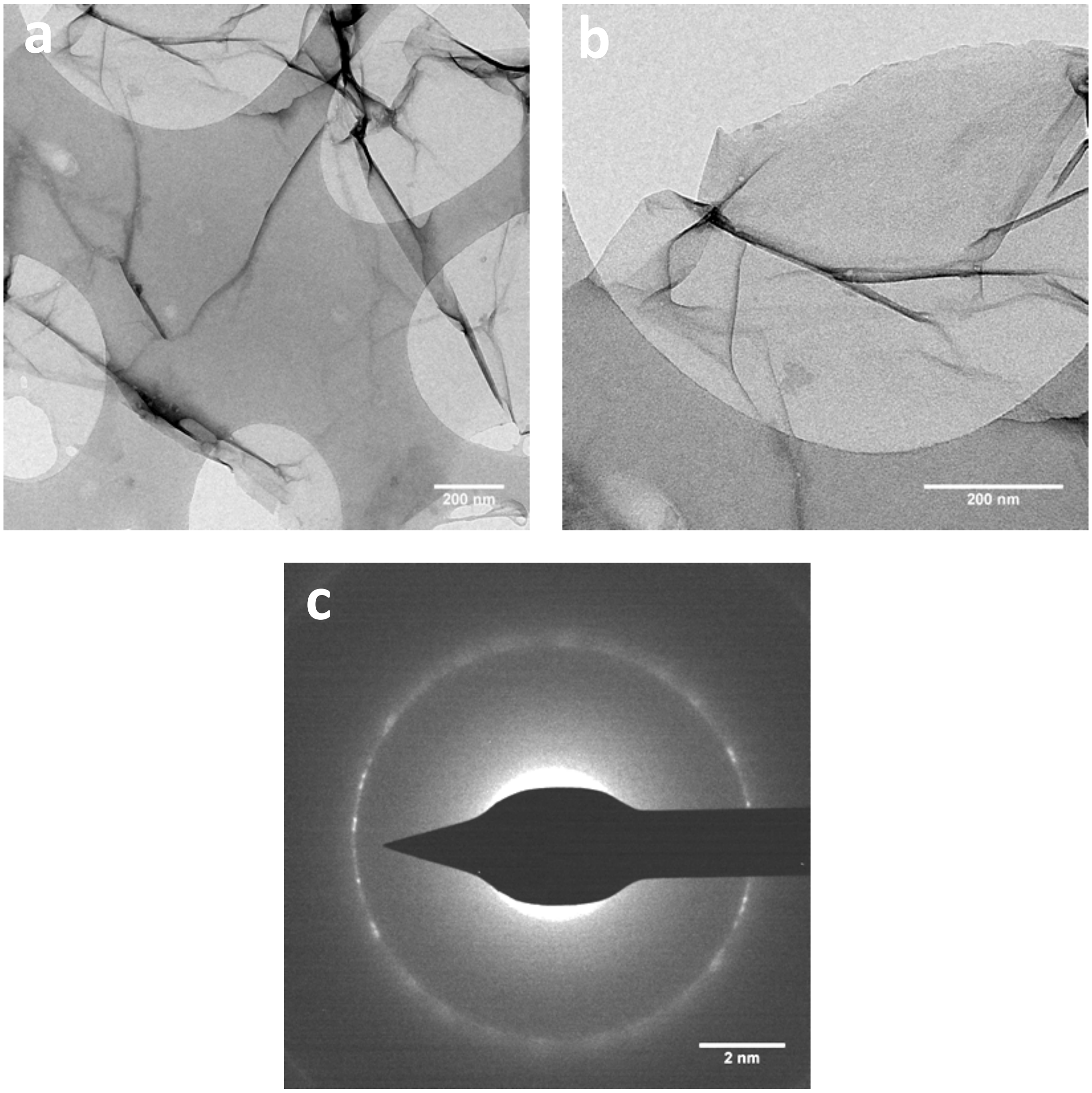

Supplementary Figure S7. High-resolution TEM images of pure RGO nanofilms. (a) And (b) HRTEM images with different magnifications, and (c) crystalline structural pattern.

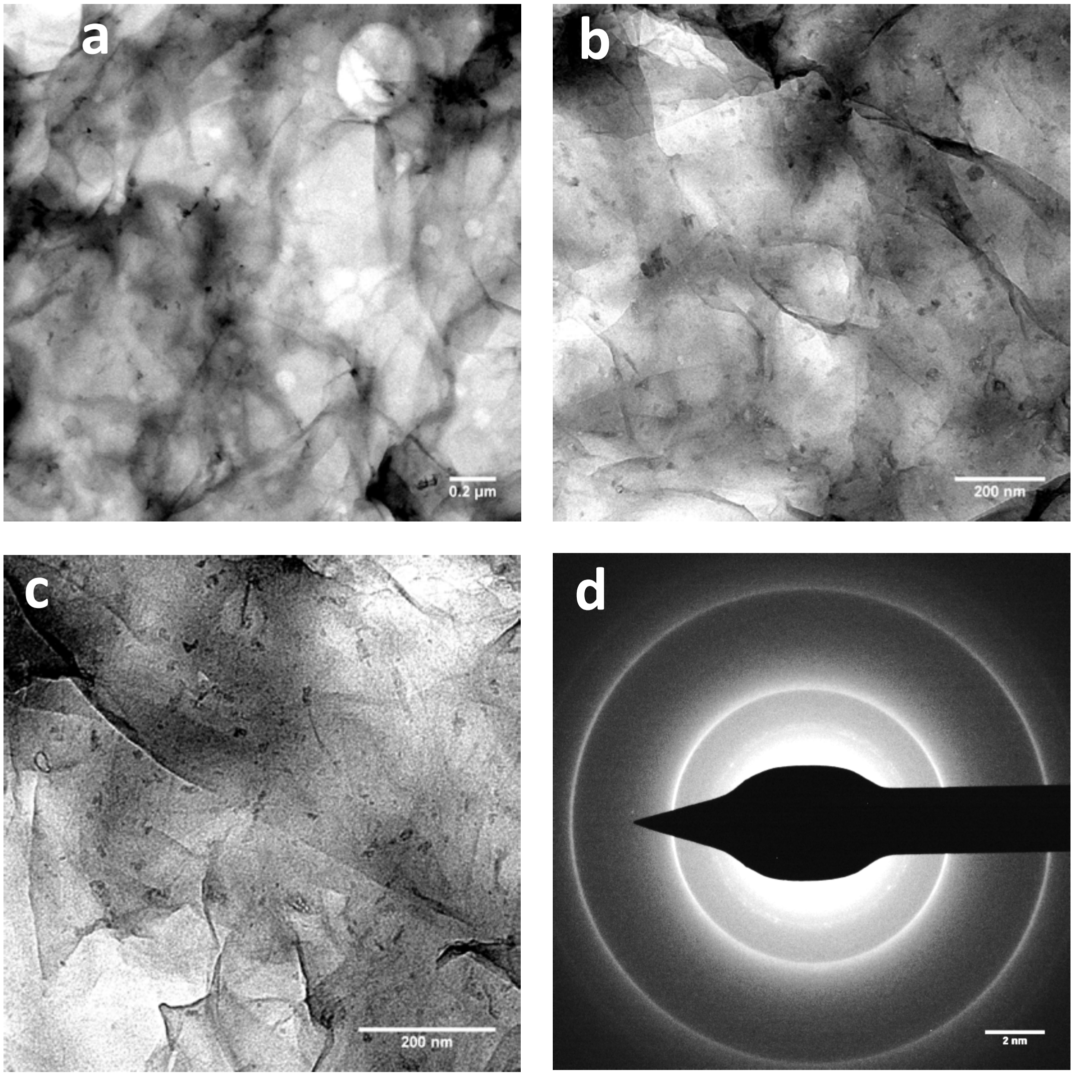

Supplementary Figure S8. High-resolution TEM images of QDs doped RGO nanofilms. (a), (b) and (c) HRTEM images with different magnifications, and (d) crystalline structural pattern.

S5. Time-resolved photoluminescence spectra

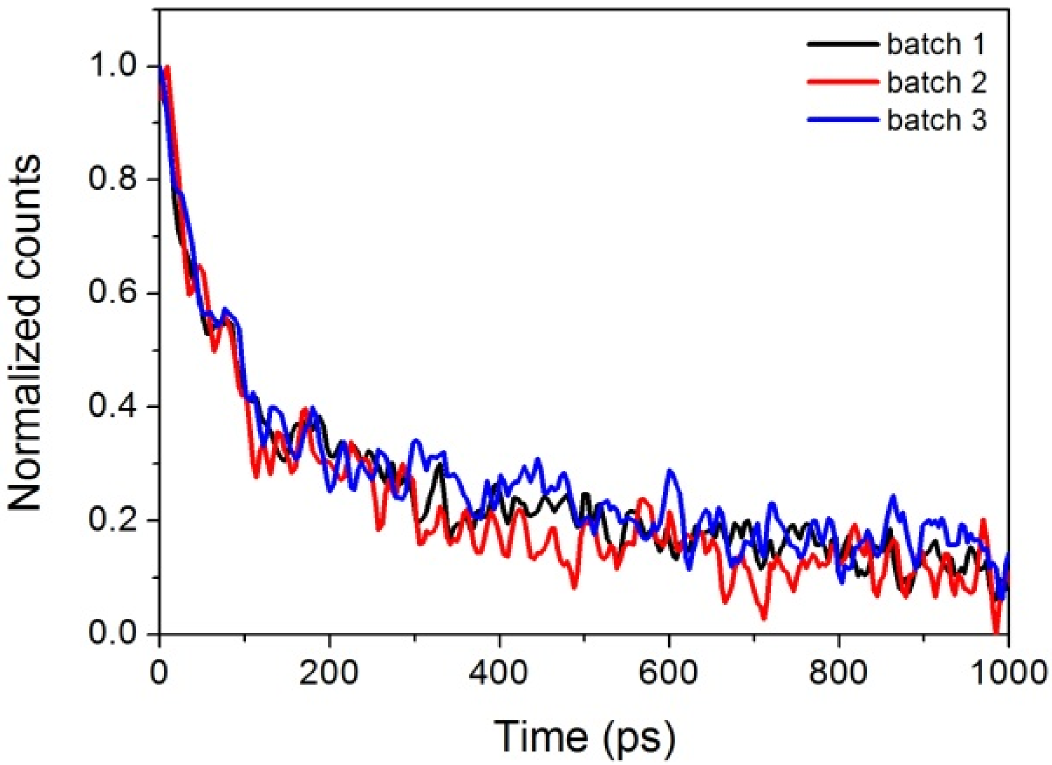

Supplementary Figure S9. Comparison of time-resolved photoluminescence spectra obtained from three batch samples prepared independently under the same experimental condition.

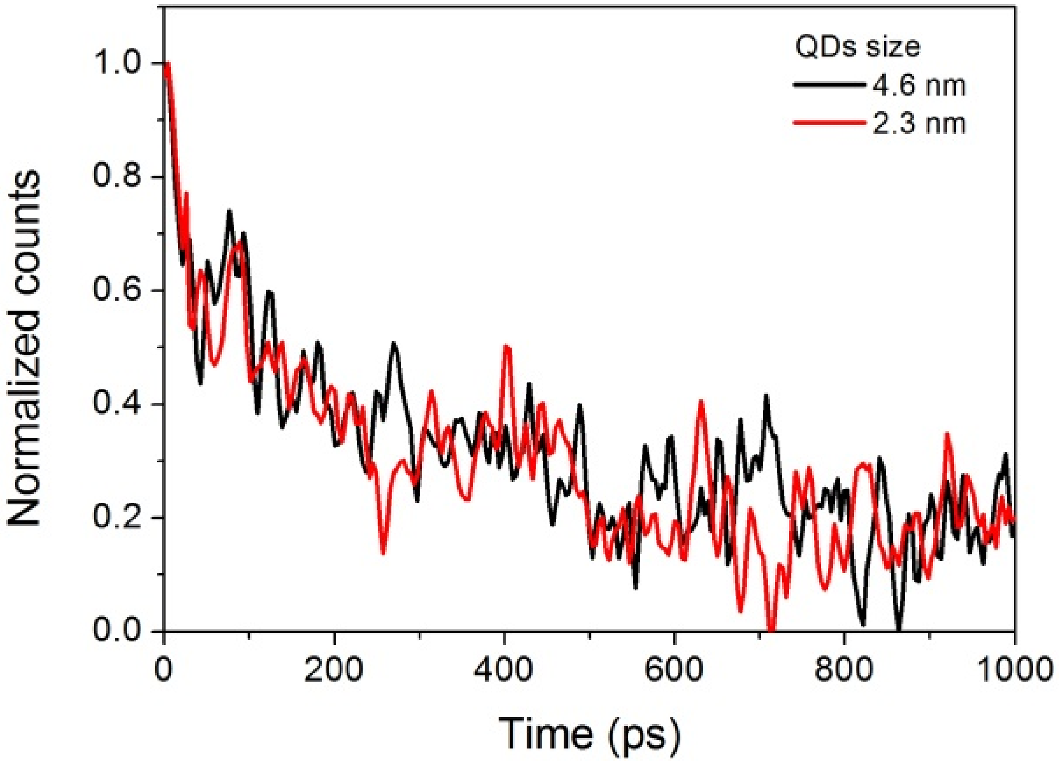

Supplementary Figure S10. Possible QD size effects on time-resolved photoluminescence spectra of QD-RGO films.

S6. Photocurrent responses of various films.

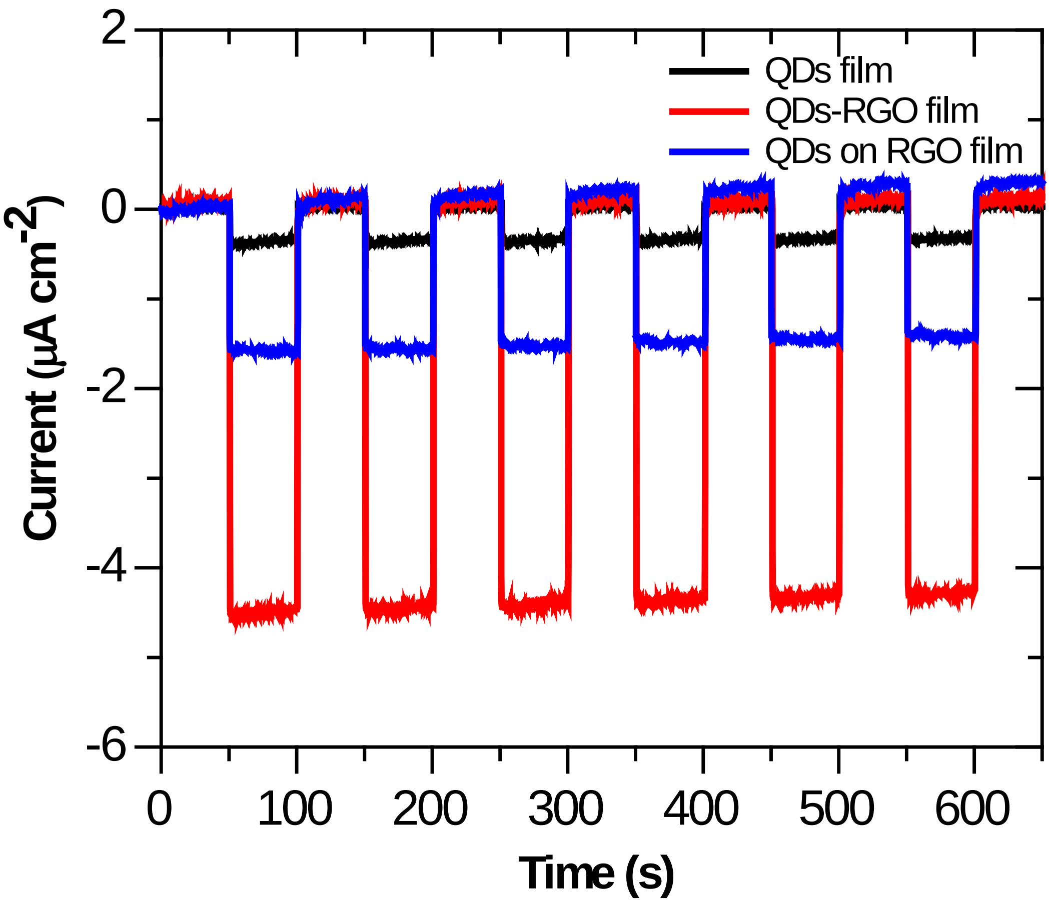

Supplementary Figure S11. Comparison of photocurrent responses of three types of films. (a) QDs film (black), (b) QDs adsorbed on the RGO film (blue) and (c) QDs sandwiched hybrid film (red).

S7. Photographs of QDs samples with different sizes.

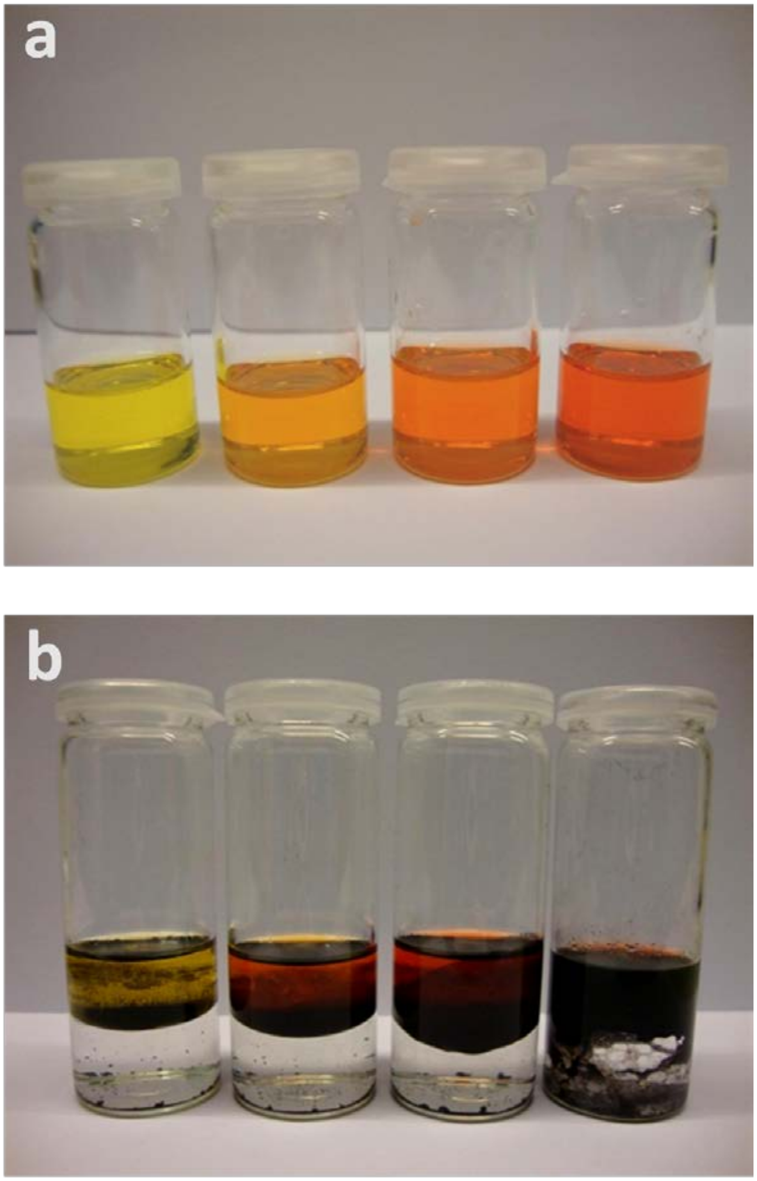

Supplementary Figure S12. Photographs of (a) QDs with different sizes in toluene, and (b) as-prepared QDs-RGO after two-phase reduction. The size of CdSe QDs in the photographs from left to right is 2.4, 2.7, 2.9 and 3.1 nm with the corresponding absorption peaks at 505, 526, 541 and 551 nm, respectively.
